# Nonadjustable state of programmable shunt valve: obstruction of middle cranial fossa arachnoid cyst-peritoneal shunt

**DOI:** 10.1186/s41016-024-00386-z

**Published:** 2024-12-26

**Authors:** Hongbin Cao, Genrui Guo, Wenjing Wu, Zhenghai Cheng

**Affiliations:** 1 Department of Neurosurgery, Hebei Children’s Hospital, Hebei Medical University, Shijiazhuang, Hebei China; 2 Department of Anesthesiology, Hebei Children’s Hospital, Hebei Medical University, Shijiazhuang, Hebei China

**Keywords:** Programmable shunt valve, Arachnoid cyst, Shunt, Shunt obstruction, Fibrosis

## Abstract

**Background:**

A nonadjustable state of the programmable shunt valve is a rare phenomenon. This case report aims to explore the cause of pressure adjustment dysfunction in a programmable shunt valve in a middle cranial fossa arachnoid cyst-peritoneal shunt patient and to underscore this dysfunction as an indicator of shunt valve obstruction.

**Case presentation:**

A child with a ruptured giant arachnoid cyst in the left middle cranial fossa presented with acute intracranial hypertension following head trauma. The initial cysto-peritoneal shunt surgery rapidly alleviated symptoms, including headaches, vomiting, and left cranial nerve palsy, stabilizing the clinical condition. However, between 20 and 24 months after the initial shunt surgery, the patient developed intermittent shunt dysfunction, experiencing recurrent headaches and vomiting, during which the programmable valve’s pressure setting had become fixed and was no longer adjustable. A second surgery was then performed to remove the existing shunt, excise the fibrotic cyst wall, fenestrate the basal cistern, and establish temporary subdural drainage. During this operation, extensive fibrosis of the cyst wall in the subdural space was discovered, forming a tough and hypertrophic fibrotic membrane that encased the cerebral hemispheres. This fibrotic material nearly filled the shunt valve chamber, causing valve obstruction and immobilizing the pressure control rod, resulting in pressure adjustment dysfunction. As the patient could not maintain stability without continuous drainage, a third surgery was ultimately necessary to place a subdural-peritoneal shunt. Five years of follow-up revealed no significant clinical symptoms, and the patient has maintained a normal life.

**Conclusion:**

Shunt valve obstruction is an underestimated cause of shunt system failure, with no current definitive method for early diagnosis. Fibrotic deposition is a primary mechanism underlying shunt valve obstruction. Pressure adjustment dysfunction in a programmable shunt valve serves as a reliable indicator of shunt valve obstruction. Further research should prioritize the treatment and prevention of shunt valve obstructions to improve outcomes in neurosurgical practice.

## Background

Arachnoid cysts in the middle cranial fossa represent a common congenital disorder of the central nervous system in children. The approach to their treatment has evolved significantly over the years and remains a subject of considerable debate. Treatment strategies have ranged from craniotomy for cyst resection to subsequent cyst-peritoneal shunt procedures, and more recently, to neuroendoscopic arachnoid cyst fenestration. Additionally, a conservative observational approach is now often adopted for asymptomatic middle cranial fossa arachnoid cysts [1–3]. Nonetheless, shunt surgery remains necessary for some young children with large arachnoid cysts in the middle cranial fossa, particularly those exhibiting significant symptoms of increased intracranial pressure [4, 5].

Shunt obstruction is one of the most common and significant complications in shunt surgery, often necessitating the adjustment or replacement of the shunt system. Existing research on the diagnosis and management of shunt obstructions has predominantly focused on the proximal and distal ends of the shunt system, while studies on obstructions within the shunt valve are notably sparse. Classical textbooks suggest that obstructions in shunt valves are much less common than those in the proximal or distal segments of the shunt [6]. However, in reality, the incidence of shunt valve obstruction is significantly underestimated, as it is one of the most intricate and critical components of the shunt system [7]. One of the primary reasons for this underestimation is the lack of clear and effective diagnostic methods for identifying shunt valve obstructions.

Shunt surgery in recent years has increasingly utilized programmable shunt valves to achieve precise control of cerebrospinal fluid (CSF) drainage [8]. In clinical practice, programmable shunt valves sometimes lose their adjustable capability, often initially presumed to be a mechanical malfunction. However, the phenomenon of pressure adjustment dysfunction has not received sufficient attention and discussion. We believe there is a significant correlation between this phenomenon and shunt valve obstruction. Establishing this correlation could provide a more reliable diagnostic criterion for clinical assessment of shunt valve obstructions.

We analyzed a case involving a child with a post-traumatic ruptured giant arachnoid cyst in the middle cranial fossa, accompanied by increased intracranial pressure, treated with a cysto-peritoneal shunt. Between 20 and 24 months postoperatively, the patient developed shunt dysfunction and pressure adjustment dysfunction of the programmable valve. This condition was later confirmed through surgery to be due to fibrotic deposition within the shunt valve. Our findings suggest that fibrotic deposition is one of the common causes of obstruction in cysto-peritoneal shunts for arachnoid cysts. Furthermore, dysfunction in the pressure adjustment of a programmable shunt valve serves as a typical and reliable indicator of shunt valve obstruction. The purpose of this case report is to provide substantial evidence demonstrating the mechanism of pressure adjustment dysfunction in programmable shunt valves and its correlation with shunt valve obstruction. This case report aims to establish a new, reliable diagnostic method for identifying shunt valve obstructions in clinical practice.

## Case presentation

**Fig. 1:**
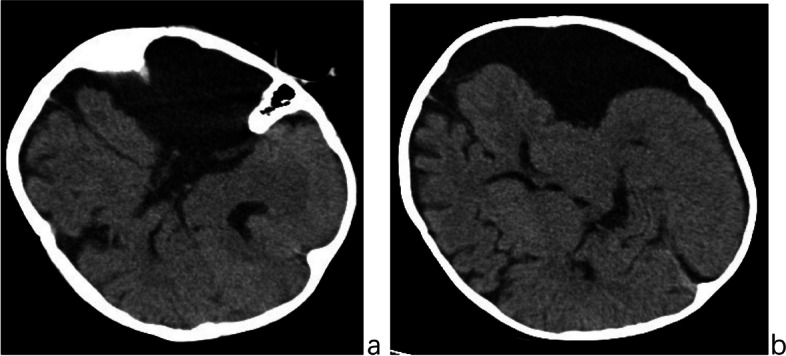
**a** and **b** Cranial CT scan showing a large left frontotemporal arachnoid cyst (**a**) and associated subdural effusion in the left frontotemporal parietal region (**b**)

**Fig. 2:**
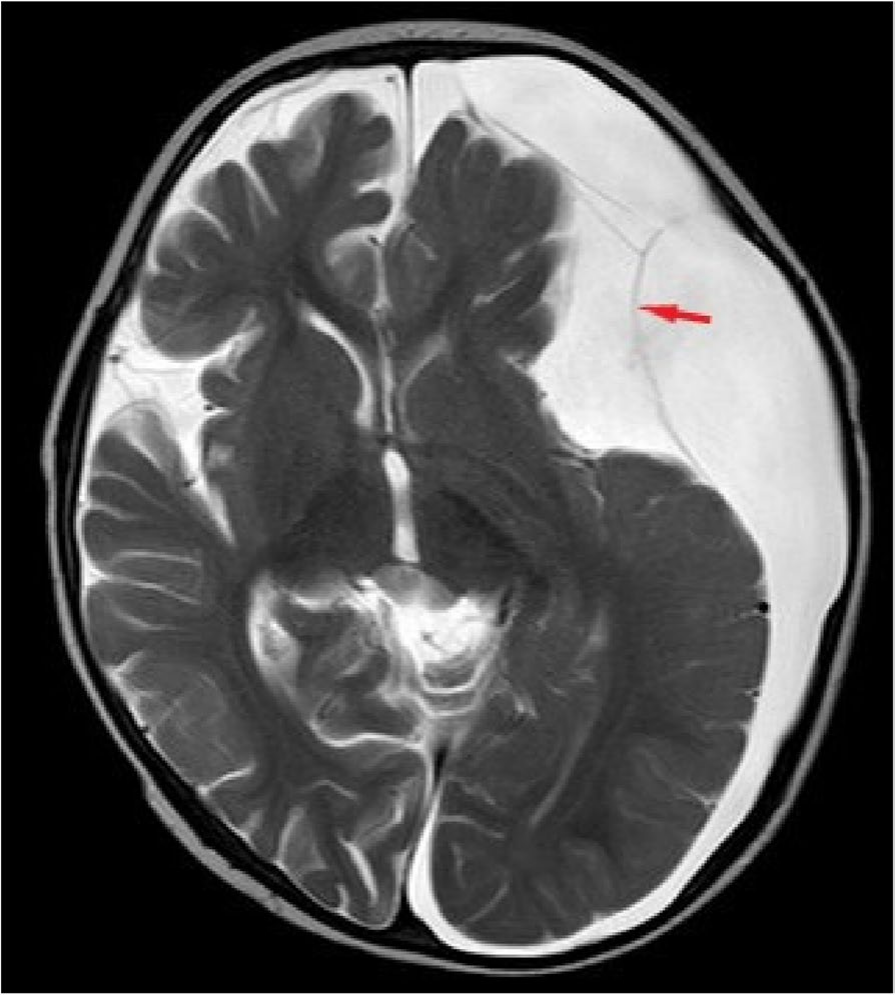
Cranial MRI demonstrating floating arachnoid signals (red arrow) within the subdural effusion, suggestive of a post-traumatic rupture of the left frontotemporal arachnoid cyst

**Fig. 3:**
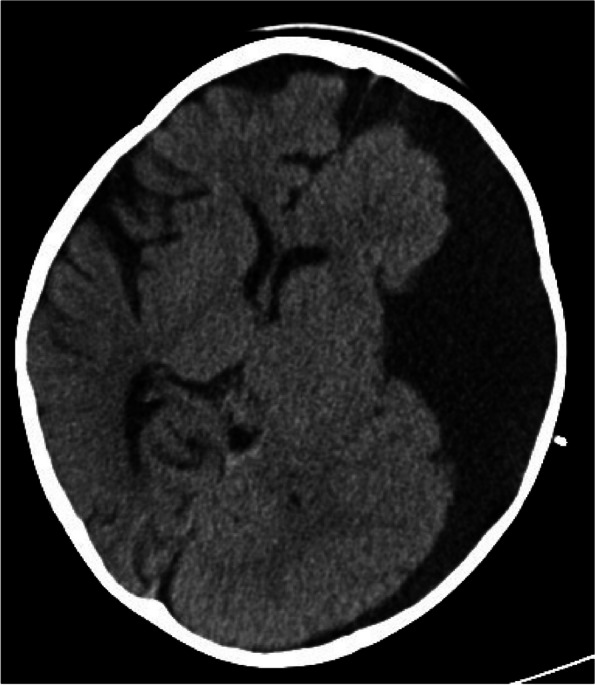
Follow-up cranial CT scan 4 days later showing increased subdural effusion with a more pronounced midline shift

A 15-month-old male child presented with poor mental responsiveness and recurrent vomiting for 8 days following a head injury sustained from a fall off a bed. Prior to the incident, the child was in good health. After a week of initial treatment and abdominal assessments at a local hospital, his condition deteriorated. A cranial CT scan conducted at our hospital revealed a large left frontotemporal arachnoid cyst accompanied by subdural effusion in the left frontotemporal parietal region (Fig. 1a & b). Subsequent cranial MRI indicated floating arachnoid signals within the subdural effusion, suggestive of a post-traumatic rupture of the left frontotemporal arachnoid cyst (Fig. 2). Despite conservative management with mannitol for dehydration, the child’s symptoms continued to worsen, leading to impaired function of the left abducens nerve. Ophthalmic examination revealed bilateral optic disc edema and engorged retinal veins, indicative of severe and sustained intracranial hypertension. Four days later, a follow-up cranial CT scan demonstrated an increase in subdural effusion with a pronounced midline shift (Fig. 3).

### First surgery

**Fig. 4:**
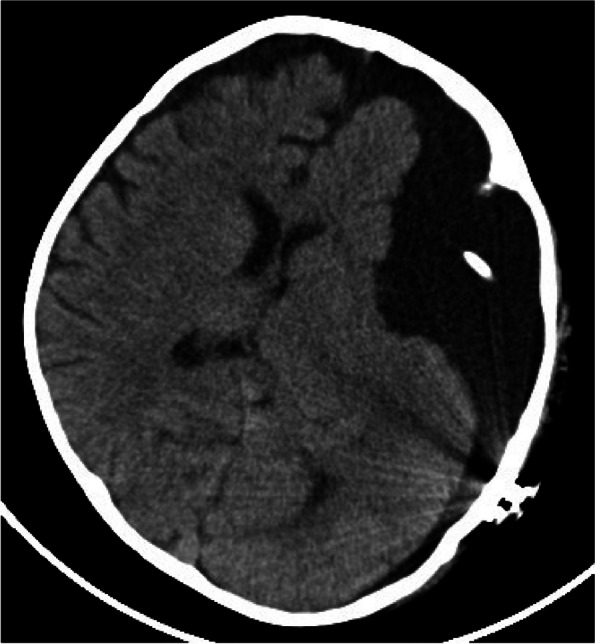
Postoperative cranial CT scan demonstrating a reduction in subdural effusion following cysto-peritoneal shunt placement

Due to severe acute intracranial hypertension and midline shift, a cysto-peritoneal shunt procedure was performed for the arachnoid cyst. Prior to surgery, the programmable shunt valve was carefully examined to confirm its pressure-regulating function and patency. Intraoperatively, extremely high CSF pressure was observed, resulting in the spontaneous ejection of CSF, which appeared clear to the naked eye. CSF analysis revealed the following: total cell counts of 30,715 × 10^6/L, with white blood cells (WBC) at 15 × 10^6/L, red blood cells (RBC) at 30,700 × 10^9/L, protein at 3.33 g/L, and glucose at 3.64 mmol/L. The elevated red blood cell count and normal white blood cell count in the CSF suggest that the subdural effusion contained blood cells due to rupture of the arachnoid membrane following trauma. This CSF profile could potentially contribute to early shunt system obstruction postoperatively. Following surgery, symptoms of abducens nerve palsy resolved within 2 weeks, and the child ceased to experience headaches or vomiting related to increased intracranial pressure. A follow-up cranial CT scan showed a reduction in subdural effusion (Fig. 4). The patient’s condition remained stable over the next 20 months.

### Second and third surgeries

Between 20 and 24 months after the initial shunt surgery, the child experienced recurrent intermittent headaches and vomiting, resulting in three hospital readmissions. The child’s condition was closely monitored, with adjustments to the treatment plan as necessary. Treatment included administering mannitol and adjusting the shunt valve pressure downward. Each time, the symptoms improved, and the child was discharged. However, with recurrent symptoms, we revised the treatment plan and opted for surgical exploration to prevent further deterioration. It was later discovered that the programmable valve’s pressure setting had become fixed and was no longer adjustable, raising concerns about shunt dysfunction. During the second surgery, the shunt system was removed, and the fibrotic wall of the arachnoid cyst in the subdural space was excised, followed by a basal cistern fenestration and the placement of temporary subdural drainage.

**Fig. 5:**
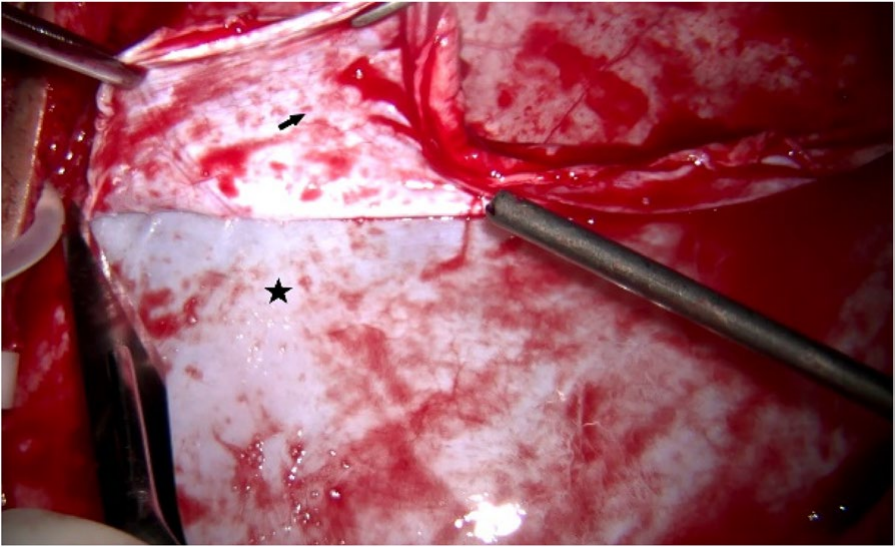
The superficial layer of the fibrotic membrane (black star) exposed following dura mater incision (black arrow)

**Fig. 6:**
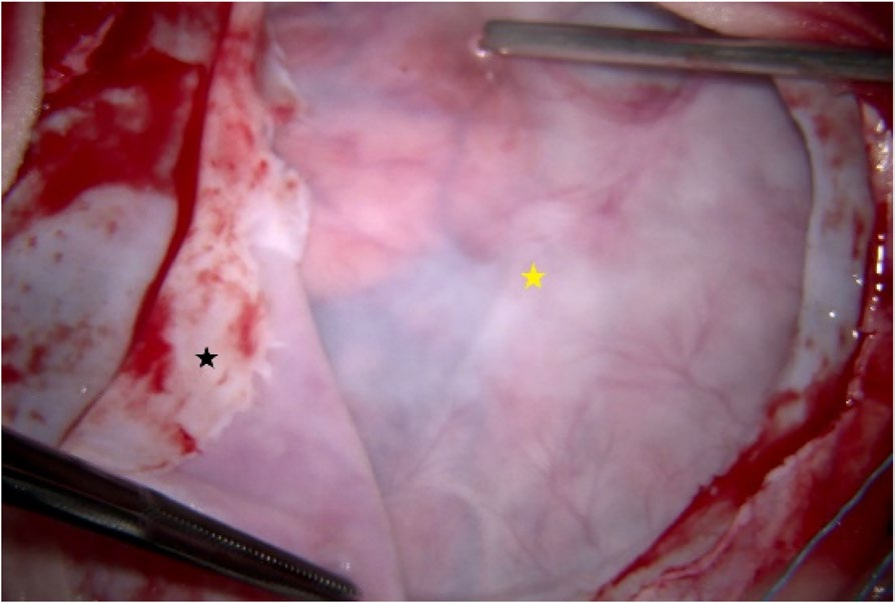
The superficial and deep layers of the fibrotic membrane formed a sealed capsule containing clear, transparent fluid. Incision of the superficial layer (black star) exposed the deep layer (yellow star) and the fluid within

**Fig. 7:**
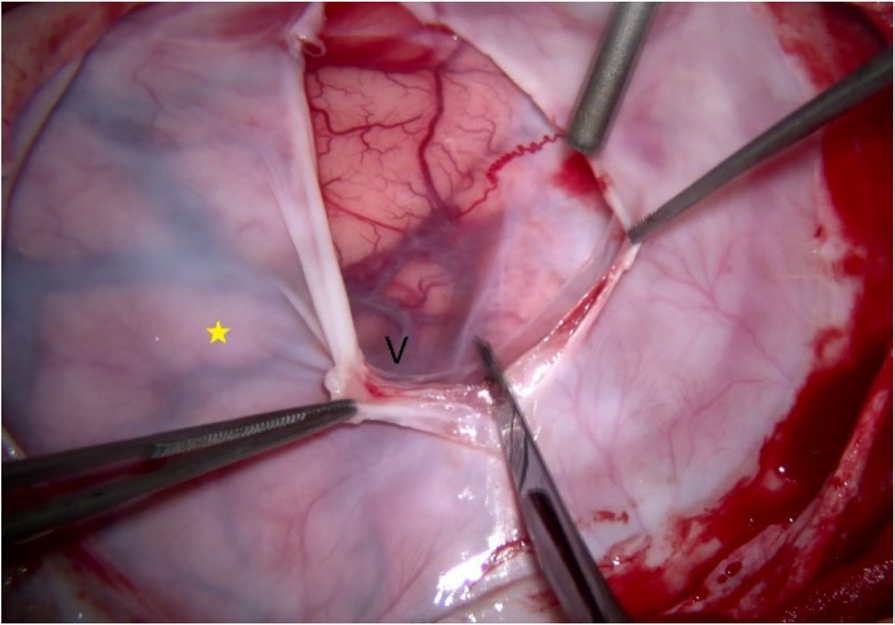
The deep layer (yellow star), which adhered to the surface of the cerebral hemisphere, required meticulous separation, particularly around the superficial cortical veins (V)

**Fig. 8:**
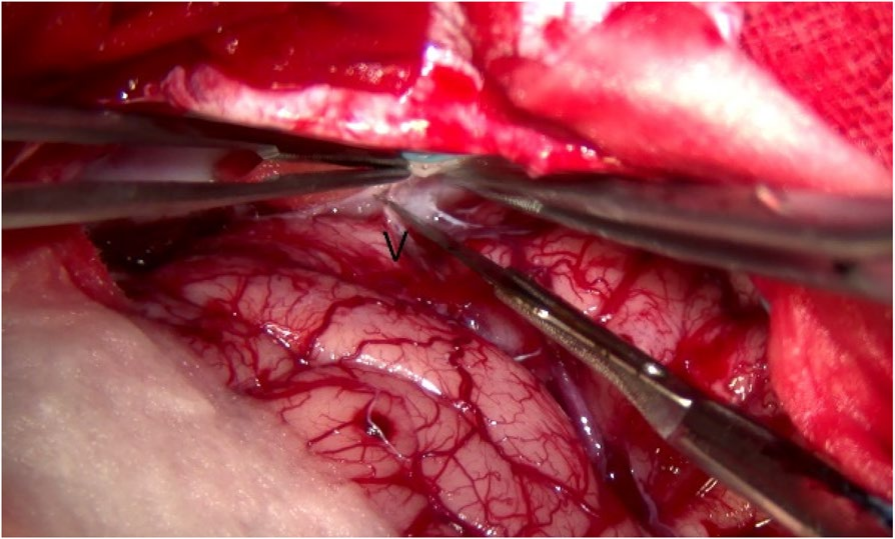
Adhesions surrounding the superficial cortical veins (V)

**Fig. 9:**
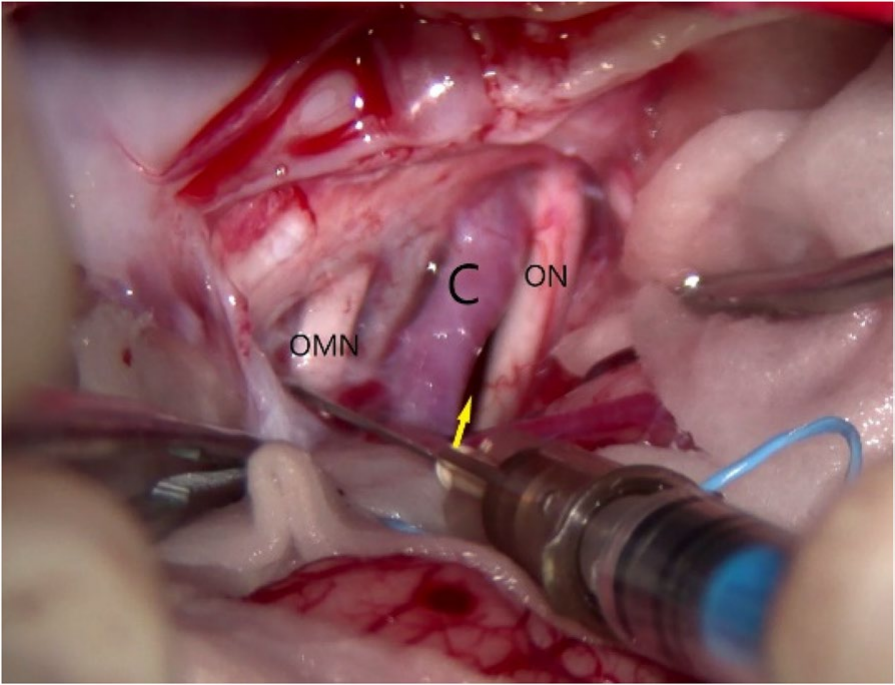
Basal cistern fenestration (yellow arrow). C, carotid artery; ON, optic nerve; OMN, oculomotor nerve

**Fig. 10:**
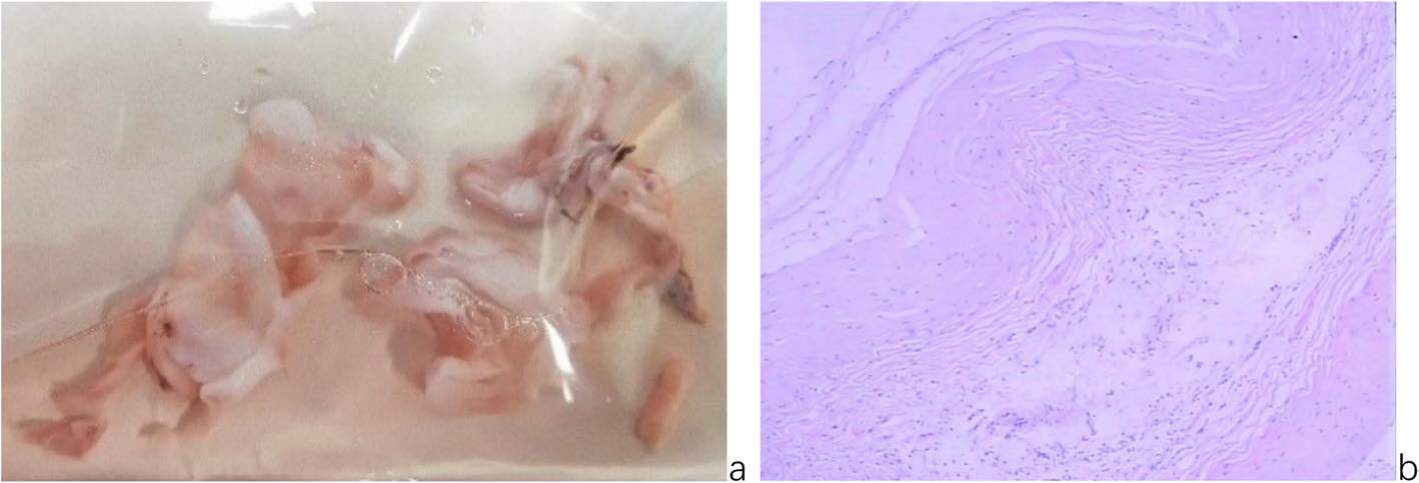
**a** and **b** Fibrotic membrane sample (**a**) and histological section (**b**), showing predominant fibrocollagenous hyperplasia

The second surgery revealed extensive and severe fibrosis of the arachnoid cyst wall in the subdural space, forming a tough and hypertrophic fibrotic membrane that encased the cerebral hemispheres. This fibrotic membrane comprised superficial and deep layers, together creating a sealed fibrous capsule filled with clear, transparent fluid. The superficial layer, which adhered to the inner surface of the dura mater, was easily peeled away. However, the deep layer, more tightly adhered to the surface of the cerebral hemisphere, required meticulous separation, especially around the superficial cortical veins, to avoid unnecessary harm to the patient. Removal of most of the fibrotic membrane successfully freed the affected cerebral hemisphere from its encasement (Figs. 5, 6, 7, 8, 9 and 10).

**Fig. 11:**
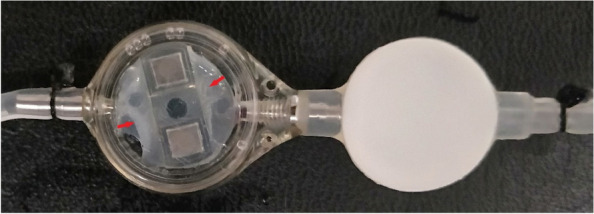
The transparent chamber of the Sophysa SM8 valve (red arrow) was filled with fibrotic deposits similar to those observed in the cyst wall, leading to obstruction of the valve and immobilization of the pressure control rod

Examination of the removed shunt system revealed no significant obstruction at either the proximal or distal ends of the shunt tube. However, the transparent chamber of the valve (Sophysa SM8) was filled with fibrotic deposits similar to those observed in the arachnoid cyst. This accumulation led to valve obstruction and immobilization of the pressure control rod, resulting in pressure adjustment dysfunction (Fig. 11).

An experimental subdural external drainage procedure was performed to drain the bloody and turbid fluid from the surgical area, aiming to assess the child’s adaptation after shunt system removal. After 1 week of intermittent clamping of the external drain and observing that the child could not maintain stability without continuous drainage, a third surgery to place a subdural-peritoneal shunt was deemed necessary.

### Follow-up

**Fig. 12:**
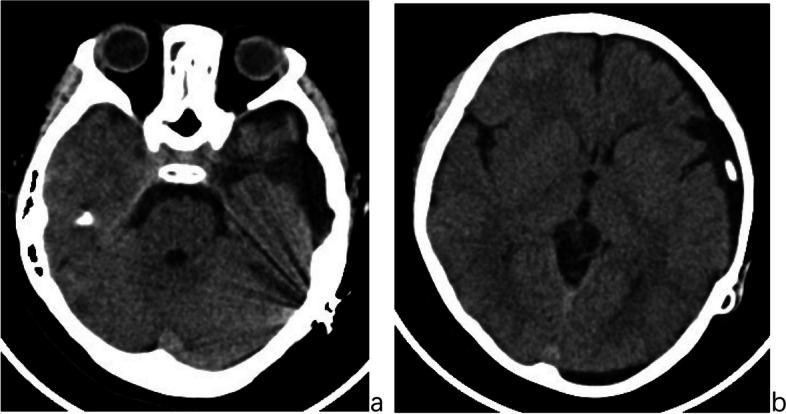
**a** and **b** Cranial CT scans obtained 5 years after the subdural-peritoneal shunt, demonstrating a decrease in both the arachnoid cyst and subdural effusion

After the subdural-peritoneal shunt, the child’s clinical symptoms resolved. Over 5 years of follow-up, he has maintained good health, attended school, and led a normal life. Follow-up cranial CT scans show stable intracranial conditions, with a reduction in both the arachnoid cyst and subdural effusion (Fig. 12a and b).

## Discussion

The treatment of arachnoid cysts in the middle cranial fossa remains highly controversial. In the early stages, microscopic resection was commonly employed [9]; however, it was often accompanied by complications such as infection, hemorrhage, and subdural effusion [10]. With advancements in shunt surgery, cysto-peritoneal shunting gradually gained popularity. In the early postoperative period, shunt surgery generally resulted in stable outcomes without the complications typically associated with microscopic surgery. Notably, the significant radiological cyst reduction achieved by shunt surgery [5] made it particularly attractive to neurosurgeons. However, over time, complications associated with cysto-peritoneal shunting began to emerge, particularly challenging issues such as shunt dependency and overdrainage syndrome. These complications led to a decline in enthusiasm for shunt procedures among neurosurgeons, with some even entirely rejecting shunt surgery. In recent years, the development of neuroendoscopic techniques has increased the favorability of neuroendoscopic fenestration for treating arachnoid cysts [11]. This approach is simple and minimally invasive, and although it shares some complications with craniotomy, these complications are generally mild and easier to manage. Importantly, neuroendoscopic surgery can avoid the issue of shunt dependency associated with shunt procedures [12]. However, some studies have noted that compared to craniotomy, endoscopic fenestration may be less thorough, with a potentially higher incidence of early complications [13]. These findings have led some researchers to reconsider and support craniotomy. Choosing the appropriate surgical approach for arachnoid cysts remains challenging. The ongoing debate highlights the complexity of treatment options. Recently, a more conservative approach has gained favor, often opting for neuroendoscopic fenestration or maintaining conservative observation when feasible [4]. However, for children with large cysts and significant symptoms of increased intracranial pressure, cysto-peritoneal shunting remains a safe, effective, and, in some cases, indispensable intervention [5]. Although the use of shunt surgeries should be minimized, they have not been abandoned in managing arachnoid cysts in the middle cranial fossa. Nonetheless, choosing shunt surgery requires confronting the inherent risk of complications [13, 14].

Shunt dysfunction primarily results from infection and obstruction. While obstructions at the proximal and distal ends of the shunt system are well-documented and frequently discussed, obstructions within the shunt valves tend to be overlooked. Diagnosing obstructions at the proximal and distal ends can be relatively straightforward with a comprehensive analysis of clinical symptoms, physical examination, manipulation of the ante-reservoir, and imaging studies. However, there remains a lack of objective methods specifically tailored to diagnose obstructions within the shunt valves [15]. These valves, particularly the programmable shunt valves, are among the most delicate components of the shunt system and are especially susceptible to obstructions from blood, cellular debris, or fibrotic deposits. Theoretically, if the shunt surgery is optimally performed, the valve is the component most likely to experience obstruction. Research, including histological and electron microscopic studies, has shown that fibrotic deposition within the shunt valve is relatively common, although the extent varies [7, 16]. In this case, the child began exhibiting recurrent symptoms such as headaches and vomiting between 20- and 24-month post-shunt, yet imaging revealed no obstructions. Initially, shunt valve obstruction was not confirmed until persistent symptoms and failure of the pressure adjustment mechanism led to surgical exploration, which ultimately diagnosed valve obstruction. This case highlights the challenges in early diagnosis of shunt valve obstructions. For patients presenting with recurring headaches, vomiting, or other signs of intracranial hypertension after shunt surgery, close monitoring and timely adjustments to the treatment plan are essential to prevent further deterioration. Various methods have been explored for diagnosing shunt system obstructions. Morgan Broggi proposed a detailed clinical diagnostic algorithm [17]. Sergej Rot investigated shuntography for diagnosing mechanical obstructions in ventriculoperitoneal shunts [18]. Ariana Adamski discussed the utility of shuntograms in predicting future shunt failures [19]. Christoph Bettag conducted in vitro testing of explanted shunt valves in hydrocephalic patients with suspected valve malfunctions, demonstrating significant alterations in flow rates [15]. Jeffrey J. Quezada reported on the reliability of radiopharmaceutical shunt flow studies for detecting CSF shunt malfunctions [20]. However, diagnosing shunt system obstructions, especially shunt valve obstructions, remains challenging. Most available research focuses on valve obstructions in ventriculoperitoneal shunts. In contrast, this case provides substantial evidence of fibrin deposition-induced valve obstruction following cysto-peritoneal shunting for an arachnoid cyst, a scenario rarely reported in the literature. This case also highlights the differences in CSF environments between the ventricular and subdural spaces, suggesting that the subdural space may be more prone to fibrin deposition-related obstructions due to its unique environment. Further research is warranted to explore the differences between ventricular and cyst/subdural shunting.

The unadjustable state of the programmable valve is uncommon and may occasionally result from a mechanical malfunction of the valve itself [21]. Reports on dysfunction in pressure adjustment of shunt valves are exceedingly rare. Uwe Max Mauer first reported this issue in 2007 [22], yet subsequent literature shows limited attention to the problem, with no studies specifically addressing its correlation with shunt valve obstruction. In this patient, surgical exploration confirmed that the dysfunction was due to a substantial accumulation of fibrotic deposits. Among the many shunt patients in clinical practice, we occasionally encounter cases where the pressure adjustment function of a programmable valve fails, suggesting that this phenomenon may not be as rare as it seems. This case study not only provides substantial evidence confirming the cause of the pressure adjustment dysfunction but also supports the notion that dysfunction in pressure adjustment of a programmable valve, often due to fibrotic deposits, should be considered a reliable indicator of shunt valve obstruction.

Regarding the treatment of shunt valve obstructions, valve replacement is typically required in most cases. However, some studies, including in vitro experiments, have actively explored alternative treatments and preventive measures for shunt system obstructions. Michael Vinzani suggested that in high-risk children with shunt system obstructions, a preventive retrograde flushing device could be used to prevent blockages [23]. Emilio Gomez-Gonzalez, through in vitro experiments, proposed that contactless ultrasonic cavitation technology could potentially be used routinely as a noninvasive, preventive cleaning procedure to reduce the likelihood of obstruction [24]. Saja Al-Saloum reported that a catheter with an N-acetyl-cysteine layer increases the wettability of the surface, thereby reducing the incidence of shunt system obstructions [25]. Currently, in terms of preventing shunt system obstructions, placing the ventricular and peritoneal segments in optimal positions can help minimize the risk. However, the only reliable method to prevent shunt valve obstructions is to improve the quality of CSF before shunting. This can be achieved through external drainage, allowing the CSF to improve before performing the shunt surgery. Nevertheless, there is ongoing debate regarding the specific CSF parameters that should be met before proceeding with shunt surgery. Additionally, the high risk of infection associated with external drainage must be carefully considered.

The rupture of an arachnoid cyst leads to the formation of a subdural effusion, which subsequently results in the formation of a tough and hypertrophic fibrotic membrane, due to an activated fibrogenic mechanism in the subdural space. Cystic fluid leaks into the potential subdural space, often accompanied by subdural hemorrhage [26, 27]. Even minimal hemorrhage can stimulate a fibrogenic response, leading to the formation of a fibrotic membrane, a process that occurs slowly. If the arachnoid cyst does not rupture, such a fibrotic membrane does not develop. Interestingly, in this case, surgery revealed a hypertrophic fibrotic membrane in the subdural space and fibrotic tissue deposits of a similar nature within the shunt valve, suggesting a common origin for both types of fibrotic tissue. This fibrotic material can dissolve in the CSF, and as the fluid flows through the shunt system, it is prone to depositing in the most delicate parts of the system, potentially leading to obstructions within the shunt valves.

In contrast, the ventricular system lacks the subdural fibrogenic mechanism observed in the subdural space. Therefore, we hypothesize that arachnoid cyst/subdural-peritoneal shunts are more prone to fibrotic valve obstruction than ventriculoperitoneal shunts. In our clinical practice, we have observed that fibrotic obstructions in ventriculoperitoneal shunt valves generally occur within 10–20 years (unpublished data). Hydrocephalus and subdural effusion often occur postoperatively in patients with complex intraventricular tumors, and these conditions can transform into one another. When managing such cases, if feasible, a ventriculoperitoneal shunt is preferred over a subdural-peritoneal shunt due to its lower risk of fibrotic obstruction.

Arachnoid cysts in the middle cranial fossa typically do not cause increased intracranial pressure; however, their rupture due to trauma can lead to an acute increase. This risk is especially significant for large cysts in infants, which are more susceptible to rupture following trauma. We hypothesize that the acute increase in intracranial pressure is primarily due to a significant volume of cyst fluid entering the CSF circulation, resulting in impaired CSF absorption. Additionally, subdural effusions alter the anatomical and physiological structures of the cerebral convexity, and the presence of subdural hemorrhage further impairs CSF absorption, simulating conditions akin to acute hydrocephalus. Consequently, a cysto-peritoneal shunt was selected for the initial surgery. During the procedure, although the CSF appeared clear, laboratory tests showed elevated levels of red blood cells and proteins, common indicators of subdural fluid following cyst rupture. While these CSF findings might seem to pose a risk to shunt function, the actual obstruction occurred significantly later, 24 months postoperatively. The slow progression of fibrotic deposition suggests that the initial abnormal CSF laboratory results did not directly impact the shunt system. Instead, the direct cause of the obstruction was identified as the long-term, gradual deposition of fibrotic material, a conclusion also supported by the literature [28].

The hypertrophic fibrotic membrane underwent pathological examination, confirming that the predominant component was fibrocollagenous hyperplasia. The transparency of the Sophysa SM8 valve made it possible to observe that it was filled with fibrotic membranes, which appeared similar to those in the subdural space. These membranes immobilized the pressure control rod, rendering the valve’s pressure-regulation feature ineffective. However, a small gap remained between the fibrotic membrane and the chamber wall, allowing CSF to still flow through this narrow passage, resulting in only partial obstruction. This phenomenon explains the child’s recurrent symptoms of increased intracranial pressure.

There are varied outcomes concerning the removal of shunts in patients with arachnoid cysts. Some patients develop shunt dependency, rendering the removal of the shunt infeasible. However, there are cases where patients have stabilized after the necessary removal of the shunt [29]. In this particular case, a trial of experimental subdural external drainage was conducted following the second surgery, but the results were disappointing, necessitating another subdural-peritoneal shunt procedure.

After the subdural-peritoneal shunt procedure, the child has been followed up for 5 years, maintaining a stable condition and good health. We hypothesize that the thorough removal of the fibrotic membrane significantly reduced the fibrogenic factors within the subdural space, thereby allowing the shunt system to maintain unobstructed function for an extended period. Of course, this hypothesis necessitates further long-term follow-up.

The primary limitation of this study is the small sample size and the lack of further research on the fibrotic deposition within the valve. For instance, scanning electron microscopy (SEM) could be employed to study the structure of the obstructed valve and the nature of the deposits in more detail. We hope that these aspects can be addressed in future research. Shunt valve obstruction is an important issue, and to date, there is no definitive method for diagnosing this condition in vivo. As for the treatment of valve obstructions, the only option currently available is valve replacement. Current research is moving towards addressing these challenges, and we hope that shunt system manufacturers will intensify their efforts to develop reliable methods for diagnosing and treating shunt valve obstructions.

## Conclusion

Shunt valve obstruction is an underestimated cause of shunt system failure, with no current definitive method for early diagnosis. Fibrotic deposition is a primary mechanism underlying shunt valve obstruction. Pressure adjustment dysfunction in a programmable shunt valve serves as a reliable indicator of shunt valve obstruction. Further research should prioritize the treatment and prevention of shunt valve obstructions to improve outcomes in neurosurgical practice.

## Data Availability

Not applicable.

## Abbreviations

CSF: Cerebrospinal fluid
CT: Computed tomography
MRI: Magnetic resonance imaging
RBC: Red blood cells
SEM: Scanning electron microscopy
